# Supplementary material for: Mutations in PCYT2 disrupt etherlipid biosynthesis and cause a complex hereditary spastic paraplegia
Source: Brain. 2019 Oct 22;142(11):3382–97. doi: 10.1093/brain/awz291 (PMC6821184; doi:10.1093/brain/awz291)
Supplement: awz291_Supplementary_Data [file awz291_supplementary_data.zip › awz291-Suppl_data/Supplementary_Data4.zip!/Supplementary information 4 - plasma lipidomics.zip!/Plasma lipidomics/Control vs Patient1/Statistical plots and data/OPLS-DA.pdf]

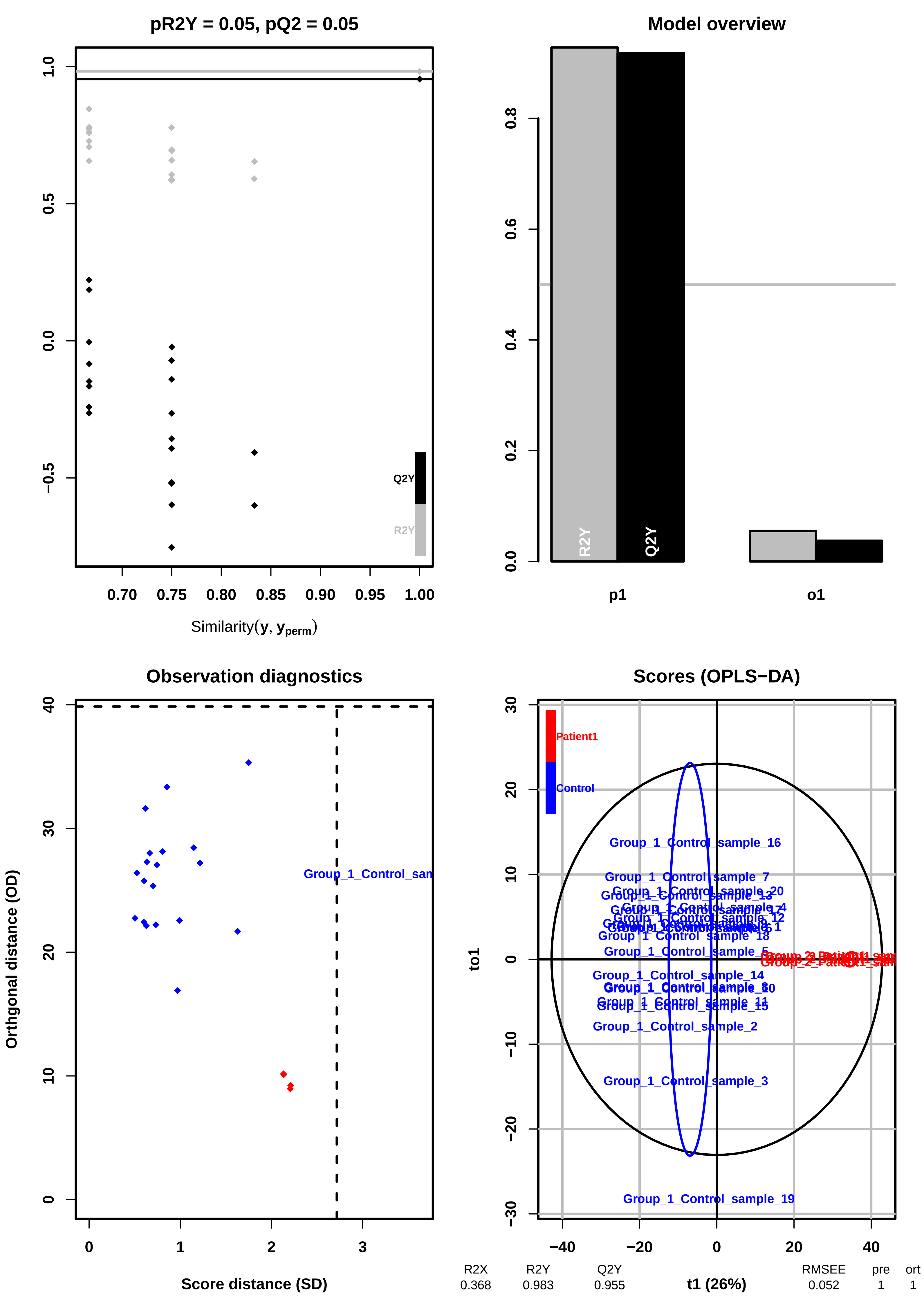

pR2Y = 0.05, pQ2 = 0.05
Similarity(y, yperm)
Q2Y
R2Y
Model overview
R2Y
Q2Y
p1
o1
Observation diagnostics
Orthgonal distance (OD)
Score distance (SD)
Group_1_Control_sam
Scores (OPLS−DA)
Patient1
Control
Group_1_Control_sample_16
Group_1_Control_sample_7
Group_1_Control_sample_18
Group_1_Control_sample_14
Group_1_Control_sample_2
Group_1_Control_sample_3
Group_1_Control_sample_19
to1
t1 (26%)
R2X 0.368
R2Y 0.983
Q2Y 0.955
RMSEE 0.052
pre 1
ort 1
